# Supplementary material for: NLGN3 autism variants have distinct functional impact on synapses and sleep behavior in Drosophila
Source: bioRxiv. 2026 Mar 27:2026.03.26.714389. Preprint. [Version 1] doi: 10.64898/2026.03.26.714389 (PMC13041802; doi:10.64898/2026.03.26.714389)
Supplement: Supplement 1 [file NIHPP2026.03.26.714389v1-supplement-1.pdf]

bioRxiv preprint doi: https://doi.org/10.64898/2026.03.26.714389; this version posted March 30, 2026. The copyright holder for this preprint (which was not certified by peer review) is the author/funder, who has granted bioRxiv a license to display the preprint in perpetuity. It is made available under aCC-BY 4.0 International license.

**Table S1: Identified 23 ASD-associated *NLGN3* variants and used bioinformatic programs to prioritize variants of interest**

| Variant (BC051715.1 transcript) | cDNA | Inheritance Pattern | Biological Sex | Allele Frequency | CADD | PolyPhen2 | SIFT | Publication |
|---|---|---|---|---|---|---|---|---|
| p.P120L | c.359 C>T | *De Novo* | Male | 8.26E-07 | 28.7 | 0.802 | 0 | Laurie et al (2025) |
| **p.R175W** | **c.523 C>T** | ***De Novo*** | **Female** | **1.65E-05** | **30** | **0.982** | **0** | **Iossifov et al. (2014)** |
| p.G406S | c.1216 G>A | *De Novo* | Female | 8.25E-07 | 26.7 | 0.023 | 0.01 | Xu et al. (2014) |
| p.R640C | c.1918C>T | *De Novo* | Male | 3.30E-06 | 25.9 | 0.761 | 0 | ClinVar (2024) |
| p.L721R | c.2162 T>G | *De Novo* | Male | Absent | 17.95 | 0.063 | 0.19 | Seth et al. (2023) |
| p.G188R | c.562G>A | Maternal | Male | Absent | 33 | 0.995 | 0 | Qin et al. (2025) |
| p.S249N | c.746G>A | Maternal | Male | Absent | 24.2 | 0.001 | 0.03 | ClinVar (2021) |
| p.I252F | c.754A>T | Maternal | Male | Absent | 28.5 | 0.946 | 0 | ClinVar (2022) |
| p.V326M | c.976 G>A | Maternal | Male | 3.31E-06 | 24.2 | 0.102 | 0.05 | Jiang et al. (2013) |
| p.V341A | c.1022 T>C | Maternal | Male | 2.31E-05 | 26.1 | 0.698 | 0 | Yu et al. (2013) |
| **p.R451C** | **c.1351 C>T** | **Maternal** | **Males (x2)** | **Absent** | **33** | **1** | **0** | **Jamain et al. (2003)** |
| p.P514S | c.1540 C>T | Maternal | Males (x2) | Absent | 26 | 0.97 | 0 | Quartier et al. (2019) |
| p.M538V | c.1612A>G | Maternal | Male (x3) | Absent | 27 | 0.998 | 0 | ClinVar (2021) |
| p.T542I | c.1625C>T | Maternal | Male | Absent | 31 | 0.962 | 0 | ClinVar (2024) |

bioRxiv preprint doi: https://doi.org/10.64898/2026.03.26.714389; this version posted March 30, 2026. The copyright holder for this preprint (which was not certified by peer review) is the author/funder, who has granted bioRxiv a license to display the preprint in perpetuity. It is made available under aCC-BY 4.0 International license.

| | | | | | | | | |
|---|---|---|---|---|---|---|---|---|
| p.D594N | c.1780 G>A | Maternal | Male | Absent | 27.5 | 0.793 | 0 | Sedlackova et al. (2024) |
| **p.R597W** | **c.1789 C>T** | **Maternal** | **Males (x3)** | **8.29E-07** | **28.8** | **1** | **0** | **Redin et al. (2014), Quartier et al. (2019)** |
| p.T632A | c.1894 A>G | Maternal | Males (x2) | 3.83E-03 | 20.7 | 0 | 0.08 | Blasi et al. (2006) |
| p.R777W | c.2329C>T | Maternal | Male | Absent | 24.8 | 1 | 0 | ClinVar (2023) |
| p.E139K | c.415G>A | Unknown | Male | 8.26E-07 | 32 | 1 | 0.02 | ClinVar (2025) |
| p.Y147H | c.439 T>C | Unknown | Male | Absent | 28.3 | 1 | 0 | Greco et al. (2025) |
| p.N216S | c.647A>G | Unknown | Unknown | Absent | 25 | 0.965 | 0 | ClinVar (2021) |
| p.A442T | c.1324G>A | Unknown | Unknown | Absent | 32 | 0.958 | 0 | ClinVar (2022) |
| p.H761R | c.2282A>G | Unknown | Unknown | 7.52E-06 | 18.56 | 0.28 | 0.02 | ClinVar (2024) |

This table summarizes all ASD-associated *NLGN3* variants published in SFARI or ClinVar databases with the year the variant was submitted. Variants selected for functional analysis are bolded. The recurrence of a variant within a family are noted in the parentheses by the biological sex. Allele frequency was obtained from gnomAD. CADD: combined annotation dependent depletion; PolyPhen2: polymorphism phenotyping 2; SIFT: sorting intolerant from tolerant

bioRxiv preprint doi: https://doi.org/10.64898/2026.03.26.714389; this version posted March 30, 2026. The copyright holder for this preprint (which was not certified by peer review) is the author/funder, who has granted bioRxiv a license to display the preprint in perpetuity. It is made available under aCC-BY 4.0 International license.

**S2 Table: Summary of *in silico* prediction tools used to determine the pathogenic effect of three ASD-associated *NLGN3* variants**

| Prediction Tools | Variant 1 | Variant 2 | Variant 3 |
|---|---|---|---|
| cDNA (BC051715.1 transcript) | c.523 C>T | c.1352 C>T | c.1789 C>T |
| Protein | p.R175W | p.R451C | p.R597W |
| Inheritance Pattern | *De Novo* | Maternally Inherited | Maternally Inherited |
| Allele Frequency | 1.65E-05 | Absent | 8.29E-07 |
| SIFT | Deleterious (0) | Deleterious (0) | Deleterious (0) |
| PolyPhen2 | Probably damaging (0.985) | Probably damaging (1) | Probably damaging (1) |
| CADD | 30 | 33 | 28.8 |
| REVEL | 0.477 | 0.767 | 0.789 |
| Alpha Missense | Ambiguous (0.5203) | Likely pathogenic (0.9417) | Likely pathogenic (0.9942) |
| MutationTaster | Deleterious | Deleterious | Deleterious |
| MutPred2 Score | 0.798 | 0.908 | 0.867 |
| MutPred2 Molecular Mechanism | Loss of intrinsic disorder, loss of B-factor, loss of ADP-ribosylation, gain of proteolytic cleavage | Altered ordered interface, altered transmembrane protein, loss of ubiquitylation | Altered ordered interface, loss of relative solvent accessibility, altered DNA binding, altered transmembrane protein |

This table summarizes the prediction tools used to determine the pathogenicity of each variant. SIFT: sorting intolerant from tolerant; PolyPhen2: polymorphism phenotyping 2; CADD: combined annotation dependent depletion; REVEL: rare exome

bioRxiv preprint doi: https://doi.org/10.64898/2026.03.26.714389; this version posted March 30, 2026. The copyright holder for this preprint (which was not certified by peer review) is the author/funder, who has granted bioRxiv a license to display the preprint in perpetuity. It is made available under aCC-BY 4.0 International license.

## S3 Table: Summary of overexpression assays performed

| | | *NLGN3*$^{Ref}$ | *NLGN3*$^{R175W}$ | *NLGN3*$^{R451C}$ | *NLGN3*$^{R597W}$ |
|---|---|---|---|---|---|
| **Motor function (Neuronal driver)** | Locomotion | ⇩ | ⇩ | ns | ⇩ |
| | Activity per waking minute | ns | ns | ns | ns |
| **Sleep behavior (Neuronal Driver)** | Sleep latency | ⇩ | ns | ns | ns |
| | Sleep duration | ⇧ | ⇧ | ns | ⇧ |
| | Sleep bout | ⇩ | ⇩ | ns | ns |
| | Bout length | ⇧ | ⇧ | ns | ns |
| **Synaptic architecture** | Bouton number (*Nlg3*$^{TG4}$ driver) | ns | ⇧ | ⇧ | ns |
| | Bouton number (neuronal driver) | ns | ⇧ | ⇧ | ns |
| | Bouton number (motor neuron driver) | ns | ⇧ | ⇧ | ns |
| | Bouton number (muscle driver) | ⇩ | ns | ns | ns |

This table summarizes overexpression assays performed. *NLGN3*$^{Ref}$ animals were compared to *LacZ* animals. *NLGN3* variants were compared to *NLGN3*$^{Ref}$ animals. Arrows indicate whether the behavior increased or decreased from their respective controls. ns = not statistical difference when compared to their respective controls

bioRxiv preprint doi: https://doi.org/10.64898/2026.03.26.714389; this version posted March 30, 2026. The copyright holder for this preprint (which was not certified by peer review) is the author/funder, who has granted bioRxiv a license to display the preprint in perpetuity. It is made available under aCC-BY 4.0 International license.

**Figure S1: *NLGN3* variants are well conserved across other *NLGN* family members.**

**A)** Evolutionary conservation of amino acids across other human NLGN family members. **B, E)** *Nlg3*$^{TG4}$ driving a G-trace reporter line was used to identify the developmental expression of *Nlg3*. **C)** anti-Elav staining labels neurons. **D)** Merging the expression of *Nlg3* and Elav-positive cells identifies that *Nlg3* is expressed in neurons during

bioRxiv preprint doi: https://doi.org/10.64898/2026.03.26.714389; this version posted March 30, 2026. The copyright holder for this preprint (which was not certified by peer review) is the author/funder, who has granted bioRxiv a license to display the preprint in perpetuity. It is made available under aCC-BY 4.0 International license.

development. **F)** anti-Repo staining labels glial cells. **G)** Merging *Nlg3* and Repo-positive cells shows some overlap between *Nlg3* and glia during development. Scale bar: 100 μm.

**Figure S2: Overexpression of the p.R175W variant alters sleep behaviors. A)** Quantification of the activity per waking minute (n≥19 flies; Welch's ANOVA with Dunnett's T3 multiple comparison test, ns $P > 0.05$, * $P \leq 0.05$, ** $P \leq 0.01$, **** $P \leq 0.0001$). **B)** Quantification of the total activity measured by video recording adult flies (n≥20 flies). **C)** Quantification of total activity in *NLGN3* overexpression models using a neuronal GAL4 driver (n≥30 flies). **D)** Quantification of activity per waking minute in *NLGN3* overexpression models (n≥30 flies). **E)** Quantification of sleep duration during lights on (n≥30 flies, Welch's ANOVA with Dunnett's T3 multiple comparison test, ** $P \leq 0.01$, **** $P \leq 0.0001$). **F)** Quantification of sleep duration during lights off (n≥30 flies). **G)** Quantification of sleep latency (n≥30 flies). **H)** Quantification of average sleep bout number during lights off (n≥30 flies, Welch's ANOVA with Dunnett's T3 multiple comparison test, ns $P > 0.05$, *** $P \leq 0.0001$). **I)** Quantification of average sleep bout length during lights off (n≥30 flies). Kruskal Wallis with Dunn's multiple comparison test, for B-D, F, G, and I; ns $P > 0.05$, * $P \leq 0.05$, ** $P \leq 0.01$, *** $P \leq 0.001$, **** $P \leq 0.0001$.

**Figure S3: *NLGN3* variants do not impair branching.** Quantification of the number of branches in larval neuromuscular junctions at muscle 6/7 of abdominal segment A4 from $y^{1}$ $w^{*}$, $Nlg3^{TG4/Null}$, $Nlg3^{Null/Null}$, $NLGN3^{Ref}$; $Nlg3^{TG4/Null}$, $NLGN3^{R175W}$; $Nlg3^{TG4/Null}$, $NLGN3^{R451C}$; $Nlg3^{TG4/Null}$, and $NLGN3^{R597W}$; $Nlg3^{TG4/Null}$ larvae (n≥22 larvae, Kruskal-Wallis test; $P = 0.0929$).

**Figure S4: Overexpressing *NLGN3* variants with neuronal drivers alter synapse morphology. A)** Representative images of type 1B boutons at muscle 6/7 of abdominal

bioRxiv preprint doi: https://doi.org/10.64898/2026.03.26.714389; this version posted March 30, 2026. The copyright holder for this preprint (which was not certified by peer review) is the author/funder, who has granted bioRxiv a license to display the preprint in perpetuity. It is made available under aCC-BY 4.0 International license.

segment A4 from overexpressing *LacZ*, $NLGN3^{Ref}$, $NLGN3^{R175W}$, $NLGN3^{R451C}$, and $NLGN3^{R597W}$ using a $Nlg3^{TG4}$ driver. **B)** Quantification of bouton number (n≥16 larvae). **C)** Representative images of type 1B boutons at muscle 6/7 of abdominal segment A4 from overexpressing *LacZ*, $NLGN3^{Ref}$, $NLGN3^{R175W}$, $NLGN3^{R451C}$, and $NLGN3^{R597W}$ using a neuronal driver (Elav-GAL4). **D)** Quantification of bouton number (n≥17 larvae). **E)** Representative images of type 1B boutons at muscle 6/7 of abdominal segment A4 from overexpressing *LacZ*, $NLGN3^{Ref}$, $NLGN3^{R175W}$, $NLGN3^{R451C}$, and $NLGN3^{R597W}$ using a motor neuron (D42-GAL4) driver. **F)** Quantification of bouton number (n≥20 larvae). **G)** Representative images of type 1B boutons at muscle 6/7 of abdominal segment A4 from overexpressing *LacZ*, $NLGN3^{Ref}$, $NLGN3^{R175W}$, $NLGN3^{R451C}$, and $NLGN3^{R597W}$ using a muscle driver (Mef2-GAL4). **H)** Quantification of bouton number (n≥19 larvae). One-way ANOVA with Sidak's multiple comparison test, ns $P > 0.05$,* $P \le 0.05$, ** $P \le 0.01$, **** $P \le 0.0001$.

**Figure. S5: Synaptic architecture is dysregulated post development. A-G)** Representative images of 7-day-old adult abdominal neuromuscular junctions (NMJ) from $y^1\ w^*$, $Nlg3^{TG4/Null}$, $Nlg3^{Null/Null}$, $NLGN3^{Ref}; Nlg3^{TG4/Null}$, $NLGN3^{R175W}; Nlg3^{TG4/Null}$, $NLGN3^{R451C}; Nlg3^{TG4/Null}$, and $NLGN3^{R597W}; Nlg3^{TG4/Null}$ flies stained with anti-CSP, a presynaptic marker. Scale bar: 10 µm. **H)** Quantification of bouton number in an adult NMJ (n≥20 flies). One-way ANOVA with Sidak's multiple comparison test; ns $P > 0.05$, *** $P \le 0.001$, **** $P \le 0.0001$.

**Figure S6: *NLGN3* variants do not alter mEJP kinetics. A)** quantification of the amplitude of miniature excitatory junction potentials (mEJP) in $y^1\ w^*$, $Nlg3^{TG4/Null}$, $Nlg3^{Null/Null}$, $NLGN3^{Ref}; Nlg3^{TG4/Null}$, $NLGN3^{R175W}; Nlg3^{TG4/Null}$, $NLGN3^{R451C}; Nlg3^{TG4/Null}$,

bioRxiv preprint doi: https://doi.org/10.64898/2026.03.26.714389; this version posted March 30, 2026. The copyright holder for this preprint (which was not certified by peer review) is the author/funder, who has granted bioRxiv a license to display the preprint in perpetuity. It is made available under aCC-BY 4.0 International license.

and *NLGN3*$^{R597W}$; *Nlg3*$^{TG4/Null}$ larvae (n≥11 larvae; Kruskal-Wallis test, ns *P* = 0.3050). **B)** Quantification of mEJP rise time (n≥12 larvae, Kruskal-Wallis test, ns *P* = 0.1965). **C)** Quantification of mEJP decay time (n≥9 larvae; Kruskal-Wallis test, ns *P* = 0.0664). **D)** Representative mEJP traces recorded from muscle 6 at abdominal segments A3 and A4 from *y w*, *Nlg3*$^{TG4/Null}$, *Nlg3*$^{Null/Null}$, *NLGN3*$^{Ref}$; *Nlg3*$^{TG4/Null}$, *NLGN3*$^{R175W}$; *Nlg3*$^{TG4/Null}$, *NLGN3*$^{R451C}$; *Nlg3*$^{TG4/Null}$, and *NLGN3*$^{R597W}$; *Nlg3*$^{TG4/Null}$ larvae.

**Figure S7: *NLGN3* rescues endocytic defects. A-F)** Representative images of boutons from *y*$^{1}$ *w*$^{*}$, *Nlg3*$^{TG4/Null}$, *Nlg3*$^{Null/Null}$, *NLGN3*$^{Ref}$; *Nlg3*$^{TG4/Null}$, *NLGN3*$^{R175W}$; *Nlg3*$^{TG4/Null}$, *NLGN3*$^{R451C}$; *Nlg3*$^{TG4/Null}$, and *NLGN3*$^{R597W}$; *Nlg3*$^{TG4/Null}$ larvae loaded with FM1-43 dye. **H)** Quantification of the FM1-43 dye uptake measured by the intensity of the dye normalized to the size of the boutons (N≥6 larvae, n=5 boutons per larva), One-way ANOVA with Sidak's multiple comparison test; ns *P* > 0.05, * *P* ≤ 0.05, * *P* ≤ 0.01, *** *P* ≤ 0.001. Scale bar: 10 µm.
